# Single capillary oximetry and tissue ultrastructural sensing by dual-band dual-scan inverse spectroscopic optical coherence tomography

**DOI:** 10.1038/s41377-018-0057-2

**Published:** 2018-08-29

**Authors:** Rongrong Liu, James A. Winkelmann, Graham Spicer, Yunxiao Zhu, Aya Eid, Guillermo A. Ameer, Vadim Backman, Ji Yi

**Affiliations:** 1 Department of Biomedical Engineering, Northwestern University, Evanston, IL 60208 USA; 2 Department of Chemical and Biological Engineering, Northwestern University, Evanston, IL 60208 USA; 3 Department of Medicine, Boston University School of Medicine, Boston, MA 02118 USA

## Abstract

Measuring capillary oxygenation and the surrounding ultrastructure can allow one to monitor a microvascular niche and better understand crucial biological mechanisms. However, capillary oximetry and pericapillary ultrastructure are challenging to measure in vivo. Here we demonstrate a novel optical imaging system, dual-band dual-scan inverse spectroscopic optical coherence tomography (D2-ISOCT), that, for the first time, can simultaneously obtain the following metrics in vivo using endogenous contrast: (1) capillary-level oxygen saturation and arteriolar-level blood flow rates, oxygen delivery rates, and oxygen metabolic rates; (2) spatial characteristics of tissue structures at length scales down to 30 nm; and (3) morphological images up to 2 mm in depth. To illustrate the capabilities of D2-ISOCT, we monitored alterations to capillaries and the surrounding pericapillary tissue (tissue between the capillaries) in the healing response of a mouse ear wound model. The obtained microvascular and ultrastructural metrics corroborated well with each other, showing the promise of D2-ISOCT for becoming a powerful new non-invasive imaging tool.

## Imaging: powerful optical imaging tool for disease diagnosis

Scientists have developed an imaging tool for measuring the structure and oxygen levels of capillaries that transport oxygen and nutrients around the body, and could lead to better diagnosis of diseases, including cancer, hypertension, and diabetes. Monitoring the microvascular network and pericapillary tissue, which lies between capillaries, is crucial as changes in these structures are manifested in several diseases. In particular, the ability to measure capillary oxygenation and the surrounding ultrastructure could provide a better understanding of these mechanisms, but is still challenging to achieve in vivo. Now, Ji Yi from Boston University and colleagues from Northwestern University in America have developed a non-invasive optical imaging system, called dual-band dual-scan inverse spectroscopic optical coherence tomography, which combines visible light and near-infrared light interferometry, allowing them to quantify capillary sO_2_, blood flow, and pericapillary ultrastructure.

## Introduction

Biological functions rely on blood vessels for oxygen and nutrient delivery. Nutrient exchange happens at the capillary level, where the small vessel diameter (~10 µm) allows for maximum contact between the red blood cell (RBC) surface and vessel wall. The pericapillary tissue (tissue between capillaries) plays a critical role in regulating capillary function through mechanical and chemical signaling pathways^1,2^. For example, angiogenesis, the growth of new blood vessels and modification of existing ones, is a complex process involving angiogenic mediators, vessel endothelial cells, the extracellular matrix (ECM), and, in the brain, neurons, and glial cells^3,4^. The ability to quantify the microvascular network and pericapillary tissue is highly sought after, since alterations to these structures are manifested in several diseases, including cancer, inflammatory processes, hypertension, diabetes mellitus, chronic kidney disease, and neurodegenerative diseases^5–9^. In particular, monitoring capillary-level oxygen saturation (sO_2_) can indirectly assess local tissue oxygenation and metabolic function. In the case of diabetes mellitus and tumor development, angiogenesis is induced by local hypoxia and can be reflected by abnormal local oxygenation levels^10,11^. Furthermore, it has been shown that the level of collagen crosslinking in the ECM, which is a nanoscale modification, can also influence angiogenesis^12^. Therefore, measuring the true in vivo nature of capillary oxygenation and nanoscale pericapillary tissue is a highly desirable goal that, to the best of our knowledge, has not yet been achieved. As a non-invasive imaging modality, optical coherence tomography (OCT) has recently shown promise in reaching this goal.

OCT is an optical imaging modality that provides cross-sectional morphology of tissue in vivo using the coherent backscattered light from a sample^13^. OCT offers a simple, practical method to image three-dimensional (3D) tissue morphology with microscopic resolution (1–10 µm) without using ionizing radiation. In addition to 3D morphological imaging, the coupling of new signal analysis and light sources has enabled OCT to obtain functional angiography with spatially resolved sO_2_, flow rate, and oxygen delivery rate (drO_2_)^14–19^. Until now, OCT has only demonstrated the ability to extract sO_2_ from larger arteriolar-level vessels and has not shown the capability of capillary-level sO_2_ monitoring. The scattering signal from a single RBC contains information about its size, orientation, and sO_2_^20^. If the size and orientation information of an RBC are unknown, the ability to decouple this information from the sO_2_ is believed to be impossible. Larger vessel cross sections contain several RBCs, resulting in an average RBC signal that reveals sO_2._ However, the capillary cross section contains only one or two RBCs, removing the ability to instantaneously average and obtain a meaningful capillary sO_2_ signal. This is problematic since the site of oxygen exchange at the capillary level cannot be properly monitored. Therefore, it is highly desirable to advance the capabilities of OCT to provide in vivo capillary-level sO_2_.

While OCT can provide microscopic details and functional information, sensing the nanoscale structures that influence microvascular alterations, such as collagen crosslinking, is challenging. Detecting nanoscale structures is beyond the resolution limit of conventional OCT. Inverse spectroscopic OCT (ISOCT) is a new method for sensing tissue ultrastructure (structural information below the imaging resolution of microscopic histopathology). ISOCT is sensitive to structures from 30 to 450 nm and can reveal valuable tissue information, such as changes to nuclear chromatin compaction and ECM crosslinking^21,22^. As a result, coupling ISOCT with OCT angiography would allow one to isolate the pericapillary space and sense the ultrastructural changes occurring during microvascular alterations.

To address the shortcomings of capillary-level sO_2_ and pericapillary ultrastructural sensing, we developed a novel optical imaging system called dual-band dual-scan ISOCT (D2-ISOCT). We show for the first time that from a single measurement using endogenous contrast in vivo, D2-ISOCT can obtain the capillary-level sO_2_, arteriolar-level blood flow rate, drO_2_, and oxygen metabolic rate (mrO_2_) functional microvascular parameters, and characterize pericapillary space morphology and ultrastructure. As an illustration of the capabilities of D2-ISOCT, we monitored the wound-healing response of a mouse model. The multi-metric quantifications of capillary sO_2_, blood flow rate, drO_2_, mrO_2_, and pericapillary ultrastructure were temporally analyzed and show D2-ISOCT has the promise to become a powerful new non-invasive imaging tool.

## Results

### D2-ISOCT imaging system

**Fig. 1:**
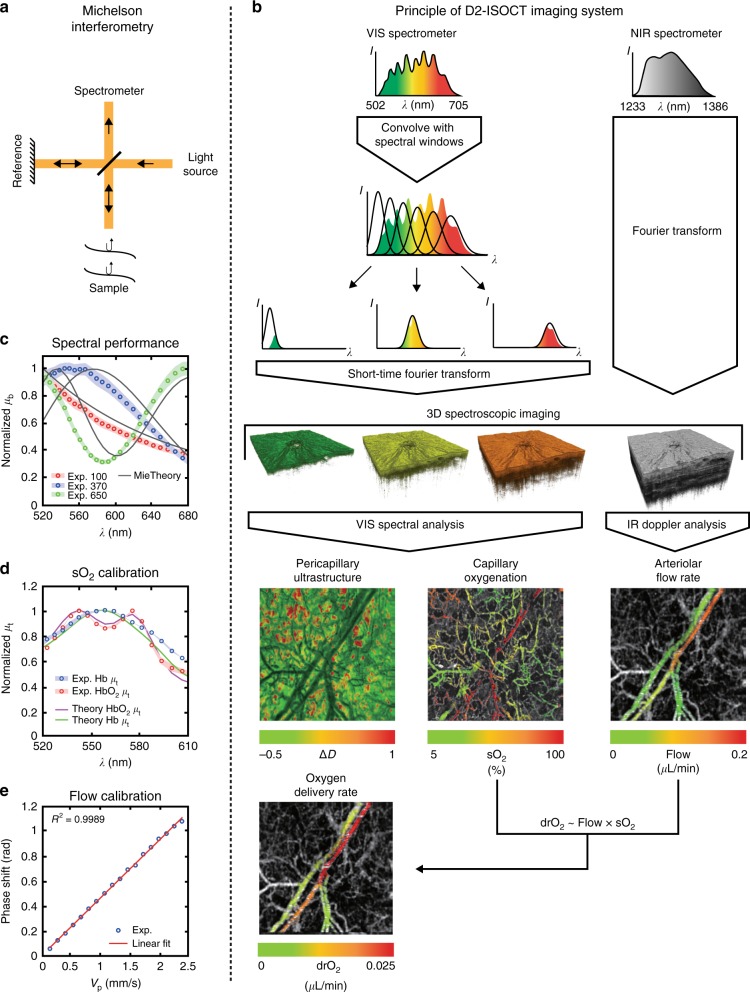
D2-ISOCT imaging system. **a** Michelson interferometry allows depth-resolved sample information to be obtained. **b** Principle behind the D2-ISOCT imaging system showing the flow from spectrometer data to the functional *en face* projections (field of view: 1.77 × 1.77 mm). **c** The backscattering spectra of beads measured with D2-ISOCT and their corresponding theoretical values from Mie theory. The bead sizes are 650, 370, and 100 nm with coefficient of variations (CV) of 3%, 3%, and 15%, respectively. **d** The sO_2_ calibration curves: the attenuation coefficients of oxygenated and deoxygenated blood measured by D2-ISOCT and calculated by Mie theory. **e** The flow speed calibration curve: the relationship between known flow speeds from a syringe pump in a microtube and phase shifts measured with D2-ISOCT

D2-ISOCT combines visible light interferometry and near-infrared (NIR) light interferometry to allow for the comprehensive quantification of capillary sO_2_, blood flow, and pericapillary ultrastructure. This study used an open-air Michelson interferometer for the visible band and a fiber-based Michelson interferometer for the NIR band. The concept of using Michelson interferometry, as depicted in Fig. 1a, is based on the interference between backscattered light from the tissue with a reference reflection to coherently gate the light from different depths in a sample. This allows the visible and NIR interferometers to obtain the 3D spectroscopic information used in computing D2-ISOCT metrics. Two broadband sources provided visible and NIR illumination, and two spectrometers individually recorded the interferogram in the visible and NIR bands. Using a dichroic mirror placed before a galvanometric mirror scanning system, the two bands were combined to allow for simultaneous scanning of each band across the sample. This is shown in the detailed schematic in Figure S1, and the additional optical components of the D2-ISOCT system are described in the Materials and methods—System setup section.

Fig. 1b demonstrates the principle behind D2-ISOCT’s spectroscopic analysis. By sweeping a Gaussian window through the visible interferometric spectrum, a short-time Fourier transform (STFT) can be applied to yield spectrally dependent OCT images. The visible angiogram allowed the hemoglobin spectroscopic signal to be analyzed separately from the pericapillary space scattering. Fitting hemoglobin light attenuation models across the 520–600 nm windows revealed sO_2_ contrast (see Supplementary Information—Microvascular Spectra and sO_2_), while fitting the tissue mass density autocorrelation function “shape factor”, *D*, across the entire visible range characterized pericapillary space ultrastructure^15,23^ (see Supplementary Information—Tissue Spectra and *D* Values).

Tissue can be characterized as a continuously varying refractive index medium with a refractive index autocorrelation function *B*_*n*_:^22^

1 $$B_n\left( r \right) = A_n\left( {\frac{r}{{l_c}}} \right)^{\frac{{D - 3}}{2}}K_{\frac{{D - 3}}{2}}\left( {\frac{r}{{l_c}}} \right)$$

where *A*_*n*_ is the amplitude of the refractive index fluctuations, *l*_*c*_ is a length scale parameter, *K*_{.}_ is the modified Bessel function of the second type, and *r* is the distance between any two points for which the autocorrelation function is being calculated. When *D* is between 0 and 3, *B*_*n*_ behaves as a power law and *D* then physically describes the fractal dimension of tissue ultrastructure. *D* values between 3 and 4 result in *B*_*n*_ having a stretched exponential shape, and as *D* approaches infinity *B*_*n*_ is a Gaussian. *D* can be related to the normalized backscattering spectra, *μ*_b_, of each imaging voxel by (2), and normalized *μ*_b_ can be related to the spectral dependent OCT A-line intensity, *I*(*k*), at each voxel by (3):^22^

2 $$\mu _{\mathrm{b}}\left( k \right)\sim k^{4 - D}$$

3 $$I^2\left( k \right)\sim \mu _{\mathrm{b}}\left( k \right)$$

where *k* is the wavenumber. Therefore, by fitting *D* to the precapillary space *μ*_b_ measured by D2-ISOCT, one can characterize ultrastructural modifications such as collagen crosslinking, which has been shown to lead to an increased *D*^22^. The tissue ultrastructural change was represented by Δ*D*, i.e., the difference from the mean *D* value.

The NIR band allowed for deeper penetration to resolve larger vessels. NIR spectrum Doppler analysis, which measured interferogram phase shifts due to sample flow, provided a large vessel flow velocity contrast that, combined with the local microvascular sO_2_, led to drO_2_. *En face* projections of capillary sO_2_, pericapillary ultrastructure, arteriolar flow rate, and drO_2_ are shown in Fig. 1b.

### System calibration and characterization

The spectral performance of the D2-ISOCT imaging system was characterized by comparing measured normalized backscattering coefficient (*µ*_b_) spectra of polystyrene beads with theoretical results calculated by Mie theory. The experimental results of beads with sizes of 650, 370, and 100 nm match well with Mie theory, as shown in Fig. 1c, with root mean square errors (RMSE) of 0.1457, 0.1243, and 0.0452, respectively. sO_2_ contrast was verified by measuring the attenuation coefficient (*µ*_*t*_) from the top 22 microns of oxygenated and deoxygenated blood samples. The blood *µ*_*t*_ spectra matched well with theoretical predictions (RMSE of 0.0449 and 0.0578 for oxygenated and deoxygenated blood, respectively) as shown in Fig. 1d and served as the calibration spectra for in vivo sO_2_. blood flow measurements with NIR Doppler analysis were calibrated by measuring the phase shifts from a microtube phantom with known flow velocities^24^. A syringe pump delivered bovine blood to a microtube with a speed range of 0.483–8.694 mm/s, covering the normal range of flow speeds in arteries, veins, and capillaries^25,26^. The Doppler angle of the microtube was 0.28 rad, giving the range of the projective flow velocities along the beam axis to be 0.13–1.22 mm/s. The results of the phantom measurements, as shown in Fig. 1e, produced a calibration curve with an excellent linear relationship between the phase shift and projective flow velocity (*R*^2^ = 0.999 to a linear model). This allowed for absolute blood flow rates to be calculated from phase shifts in vivo.

### Functional and ultrastructural imaging

**Fig. 2:**
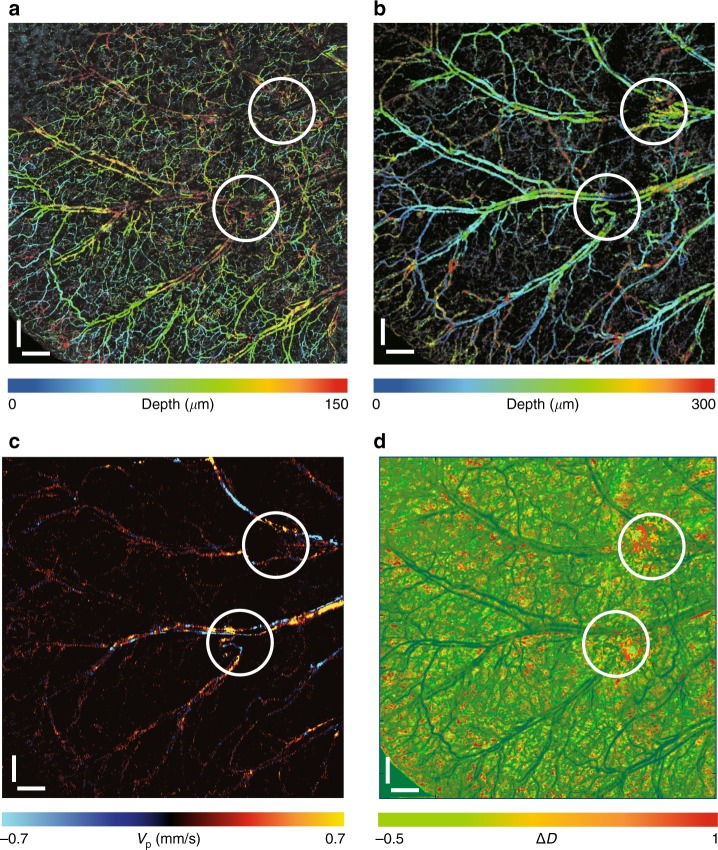
D2-ISOCT functional imaging of a SKH1-Elite hairless albino mouse ear 36 days after ear punch. **a** The *en face* projection of visible microangiography, with a depth of 0–150 μm denoted by different colors. **b** The *en face* projection of NIR microangiography, with a depth of 0–300 μm denoted by different colors. **c** The *en face* projection of blood flow velocities projected along the laser beam axis. The blue and yellow colors indicate blood flow directions running away from and toward the beam axis, respectively. **d** The *en face* projection of Δ*D* averaged along depth, 14 days after the biopsy punch (in the white circles) and 36 days after the biopsy punch (in the surrounding areas). The dark green areas indicate blood vessels of the mouse ear. The projection views in **a**–**d** are a 4 × 4 mosaic of scans. White circles in **a**–**d** indicate the regions around the wound. Scale bars: 0.5 mm

To demonstrate D2-ISOCT’s capabilities, the wound-healing process in a mouse model was monitored. Wounds were introduced into a mouse ear using a 0.35 mm biopsy punch. Large field-of-view (FOV) images (4 × 4 mosaics) of microangiography, blood flow, and ultrastructural properties are shown in Fig. 2. The images were taken from a completely healed ear, with white circles indicating two initial puncture wounds. As shown in Fig 2a, b, the visible microangiography clearly displays capillary networks, while the NIR microangiography reveals dermal arterioles and venules. A higher density of disorganized microvasculature at the wounded areas is shown in Fig. 2b, indicating angiogenesis during the wound-healing response. Blood flow velocity, which is shown in Fig. 2c, was calculated by averaging the projective blood flow velocity (*V*_p_) along the beam axis over depth. The decreased *V*_p_ in the wounded areas of Fig. 2c reflects microvascular remodeling during wound healing, corresponding well with the angiogenesis shown in Fig. 2b. Apart from neovascularization, wound healing also involves the formation of granulation tissue. To detect this formation, we used Δ*D* to quantify the ultrastructural properties of the pericapillary space. Δ*D* was calculated from the dermal layer, approximately 90–200 μm from the skin surface for a mouse ear^27^, where granulation tissue is formed during the wound-healing process^28^, as shown in Figure S6. It is clearly reflected in Fig. 2d that the wounded pericapillary space of the dermis has a higher Δ*D*.

### Longitudinal monitoring of wound healing with D2-ISOCT

**Fig. 3:**
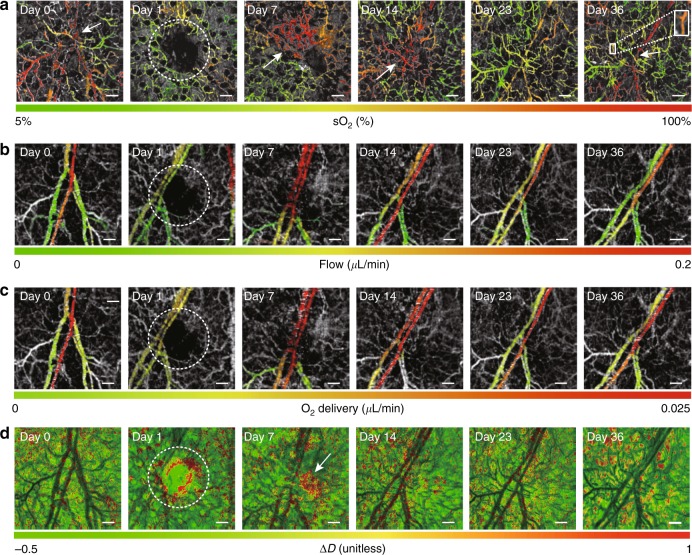
Microvascular sO_2_, blood flow rates, drO_2_, and tissue ultrastructural properties of a SKH1-Elite hairless albino mouse ear during wound healing on day 0 (no wound/control), day 1 (wound made), day 7, day 14, day 23, and day 36 post biopsy punch. **a** *En face *projections of sO_2_ microvasculature during wound healing. The white arrows in day 0 and day 36 indicate the bifurcations of a pair of blood vessels. The white arrows on day 7 and day 14 indicate significantly increasing sO_2_ in the wounded area. The white box on day 36 crops a capillary segment for which sO_2_ was calculated. **b** *En face* projections of blood flow rates during wound healing. **c**  *En face* projections of drO_2_ during wound healing. **d** *En face* projections of Δ*D* during wound healing. The white arrow on day 7 indicates an increase in Δ*D*. The white dashed circles indicate the regions around the wound in **a**–**d**. Scale bars: 200 μm

Tissue remodeling was longitudinally quantified with D2-ISOCT metrics (microvascular sO_2_, blood flow rate, drO_2_, and Δ*D*) up to 36 days after the ear punch, as shown in Fig. 3. The complete set of D2-ISOCT metrics from eight different dates, including sO_2_ measurements of dermal arterioles and venules before and after the ear punch, is shown in Figure S7. Ear punches were performed on day 1 and are marked by the dashed white circles in Fig. 3.

The sO_2_ contrast from an arteriole and a venule could roughly be distinguished at the bifurcation in Fig. 3a, day 0 (no wound/control), indicated by the white arrow. When the wound was made at this bifurcation, skin tissue was removed and the major blood vessels at this branch were disrupted. This led to local ischemia and a lower sO_2_, as shown in Fig. 3a, day 1. As the wound began, to heal there was a rapid increase in the microvascular sO_2_, as noted by the white arrows on Fig. 3a, day 7 and day 14. As the wound progressively healed, the local oxygenation level gradually returned to a state similar to that of day 0. Furthermore, in Fig. 3a, day 36 the same pair of arteriole and venules seem to reestablish their day 0 sO_2_, as noted by the white arrow. Single capillary sO_2_ sensitivity was obtained by averaging the spectra of a capillary segment over nine time points to obtain meaningful hemoglobin oxygenation spectra. The sO_2_ of a single capillary is demonstrated by the white box blow up in Fig. 3a, day 36, which had an inner diameter of ~18 µm and length of 170 µm. The segment had a sO_2_ of 72.3 ± 2.9%, which is different from the sO_2_ calculated by averaging the signals of the local capillary network, 58.5 ± 4.3%. The discrepancy in these two calculations could be due to the spatial sO_2_ heterogeneity of the capillary network.

Longitudinal blood flow rates were calculated by multiplying a vessel’s displayed cross-sectional area with its corresponding projective flow velocity. The validity of in vivo flow rates can be supported by a bifurcation flow calculation. On Fig. 3b, day 36 the flow rate before the branch (0.127 ± 0.012 μL/min) approximately matches the sum of the flow rates of the downstream branches (0.085 ± 0.004 μL/min + 0.029 ± 0.012 μL/min = 0.114 ± 0.016 μL/min), indicating reasonable in vivo performance. It can be seen in Fig. 3b that the flow rates of vessels could be clearly distinguished. The flow rates drastically dropped on day 1 and then increased on day 7. Once the wound was completely healed on day 36, the flow rate returned to the control levels. A correlation between flow rate and capillary oxygenation could be seen across all days, since flow rate and capillary oxygenation increased and decreased together, as shown in Fig. 3a, b.

**Fig. 4:**
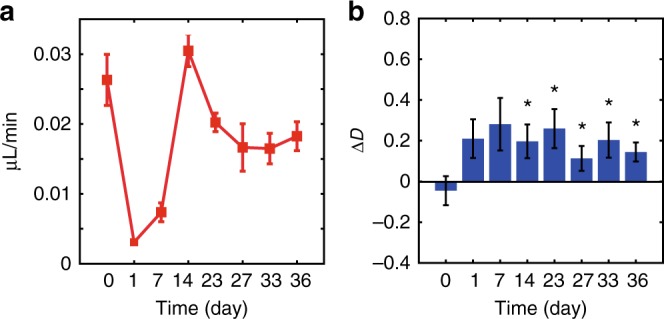
Changes in Δ*D* and mrO_2_ during wound healing. **a** The mrO_2_ calculated from the drO_2_ difference of the arteriole and venule of a SKH1-Elite hairless albino mouse ear during wound healing on day 0 (no wound/control), day 1 (wound made), day 7, day 14, day 23, day 26, day 33, and day 36 post biopsy punch. **b** A comparison of Δ*D* calculated from the dermal skin of a wound during the healing process. The histograms show the results averaged over five or six independent wounds. All statistical values are compared with the values of day 0. For day 0, day 7, and day 27, *n* = 5; for day 1, day 14, day 23, day 33, and day 36, *n* = 6.The depth range to calculate Δ*D* was 90–200 µm from the skin surface. Error bar = SEM. *p < 0.05 (the *p* values of day 7–day 36 are 0.055, 0.038, 0.023, 0.040, 0.038, and 0.037, in order)

drO_2_ was estimated using the sO_2_ of the deeper arterioles and venules. The trend of the drO_2_ matched that of the sO_2_ and flow rates. As seen in Fig. 3c, drO_2_ dropped when the wound was induced, increased during wound healing, and returned to a control-like state on day 36. We also estimated the mrO_2_ by calculating the drO_2_ difference between the arteriole and the venule. The mrO_2_ follows the same tendency as drO_2_. mrO_2_ dropped when the wound was induced, peaked during the healing process, and finally returned to a level slightly lower than the control on day 36, as shown in Fig. 4a.

During the wound-healing process, Δ*D* of the dermal pericapillary space increased. An increase in Δ*D* (indicated by the white arrow) can clearly be noted in Fig. 3d on day 7. This increase in Δ*D* during the wound-healing process is supported by a statistical analysis on the wounded areas. As shown in Fig. 4b, from day 14 to day 36 there was a significant difference (*p* < 0.05) between the control and wounded area Δ*D*s.

## Discussion

We developed a novel optical imaging system called D2-ISOCT, which enables in vivo multi-metric quantification using endogenous contrast without ionizing radiation. It was demonstrated that from a single D2-ISOCT measurement, the capillary sO_2_, arteriolar-level blood flow rates, drO_2_, and mrO_2_, as well as the pericapillary space ultrastructure, could be quantified. Using these metrics, a microvascular niche of the wound-healing process in a mouse ear was monitored over time. During the wound-healing process, sO_2_, blood flow rate, drO_2_, and mrO_2_ initially decreased due to microvascular damage and were then observed to have increased 1 week after injury, a physiologic response consistent with the intensive oxygen and nutrient demands of the healing process. Once the wound was fully healed, the microvascular metrics returned to a state similar to the control. The pericapillary space ultrastructure, quantified by Δ*D*, increased during wound healing, indicating that the scar tissue had a higher ultrastructural fractal dimension than non-perturbed tissue.

Microvascular metrics corroborated well with each other. The low oxygenation levels on day 7 could be attributed to low flow rates, which would result in less oxygen being supplied to the area, and thus lower the mrO_2_. Increased flow rates should result in an increase in the supply of oxygenation to the wounded area, which is supported by the increase in sO_2_ on day 7. In addition to the bifurcation flow calculation, the in vivo flow performance of D2-ISOCT is supported by the observation of a similar flow trend during a mouse wound-healing model from a previous study^29^. D2-ISOCT achieved capillary sO_2_ sensitivity by spatially averaging the visible spectrum to obtain local capillary sO_2_ or temporally averaging the visible spectrum (over approximately 170 s) to obtain single capillary sO_2_. The limitation of this current work is the time required to obtain single capillary sO_2_. Our simulations show that by moving to the recently developed 250 000 lines/s spectrometers and increasing sample power, this time frame can be drastically reduced.

The longitudinal monitoring of the pericapillary space ultrastructure of five to six wound sites revealed a statistically significant (*p* < 0.05) difference in the Δ*D* between the post-wounded and pre-wounded sites. Furthermore, as the wounds healed, the Δ*D* was sustained instead of gradually diminishing. This increase is most likely due to the formation of granulation tissue, which occurs during the wound-healing process^28,30^. Granulation tissue contains highly crosslinked collagen, which has previously been reported to result in an increase in *D*^22^. Hence, it is expected that the wounded area Δ*D* would increase from its non-perturbed state. This could be explained by the granulation tissue being replaced by a relatively acellular and permanent scar with a similar mass fractal dimension to the granulation tissue^30,31^. The variations in Δ*D* could be attributed to different healing speeds among wounds. Furthermore, the faster the wounds healed, the sooner a higher Δ*D* was observed, suggesting earlier collagen deposition and crosslinking.

Pioneering OCT sO_2_ work showed values ranging from 25 to 73% in a mouse dorsal window for arteriolar-level vessels^19^, which is a smaller range than that noted on day 0 in Fig. 3. However, the capillary sO_2_ values for the mouse ear are in a reasonable range in comparison with previously measured capillary sO_2_ using photoacoustics^32^. The sO_2_ value range in this work is larger than those derived from previous OCT measurements because of D2-ISOCT’s ability to measure smaller vessels through the utilization of spatial and temporal averaging. Smaller vessels contain fewer RBCs and should therefore have a larger range of sO_2_ values than vessels with sizes on the order of 10’s of microns. Furthermore, previous methods have only considered absorption in the blood attenuation model and have measured the spectra from depths just immediately below vessels^19^. Our method presented herein considers scattering and absorption in the blood attenuation model and accounts for the incident light intensity on the vessel by taking the difference in the spectra from above and below the vessel. These two advances should result in more accurate measurements of sO_2_ values since the complete attenuation model is considered and light attenuation from tissue above the vessel is accounted for.

In extracting the attenuation coefficient, *μ*_*t*_, from blood vessels for sO_2_ calculation, we assumed the accumulative attenuation of light by nonvascular tissues to be negligible compared to the attenuation of light by blood. Specifically, the backscattering coefficients from tissue above and below capillaries were approximated to be the same, such that the hemoglobin-related spectral contrast can be revealed when taking their difference, as in Eq. (6) of Materials and methods—Data processing for microvascular sO_2_ measurements. Although in principle the form of *μ*_*t*_ can change with depth depending on the scatterers and absorbers present, the above assumption works well when sufficient averaging is performed, particularly in the spatial domain, as shown in Figure S9. While the local scattering coefficient may be quite unpredictable, the averaging process smoothed the spectra and revealed the hemoglobin oxygenation contrast. Furthermore, since the spectra of blood vessels were obtained by taking the contrast immediately above and below capillaries according to the angiography mask, the accumulated attenuation through the surrounding nonvascular tissue was reduced.

The dual-wavelength bands of D2-ISOCT simultaneously provide important complementary information to offer synergistic insight into tissue functionality. While the visible light channel offers quantification of sO_2_ and ultrastructural properties, the NIR channel is complimented with better penetration to quantify the underlying blood flow, as shown in Figure S11. It can be seen in Figure S5 that the NIR deep penetration also allowed the imaging of vessels, which could not be seen in the visible channel. Additionally, this study lays the ground work for other research applications and clinical implementation of the technology.

D2-ISOCT provides a powerful new toolbox for existing OCT imaging applications in both laboratory and clinical settings. In the brain D2-ISOCT may allow researchers to probe the functional vascular changes that occur during organ development or disease progression^33,34^. In the clinic, integrating D2-ISOCT into endoscope measurements might further enrich the contrast for OCT endoscopic imaging^35–38^. For example, in the colon, it has been shown that the D2-ISOCT metrics *D* and the vessel organization can be used in evaluating colorectal cancer risk^39,40^. Additionally, it is well known that changes in the local oxygenation and metabolic rate are hallmarks of tumorigenesis^41^. Therefore, clinical endoscopic D2-ISOCT imaging systems may provide novel insights and diagnostic information.

To summarize, we have demonstrated for the first time in vivo single capillary oximetry and pericapillary ultrastructural sensing using a novel imaging system called D2-ISOCT. We obtained measurements of sO_2_, blood flow rate, drO_2_, and tissue ultrastructural properties during the wound-healing process in a mouse ear. Our D2-ISOCT system successfully detected microvascular alterations and tissue remodeling during wound recovery, indicating the potential of D2-ISOCT as a powerful new imaging tool. Future plans will aim to reduce single capillary sO_2_ measurement time and explore optical clearing agents to aid in deep sO_2_ calculations.

## Materials and methods

### System setup

Figure S1 depicts the benchtop D2-ISOCT imaging system. The system utilized a supercontinuum light source (SuperK Extreme, NKT Photonics; spectral range: 480–2200 nm) and a super luminescent diode (SLD1325, Thorlabs; spectral range: 1250–1380 nm) for visible and NIR illumination, respectively. To allow for a sufficient dynamic range across the spectrum, the supercontinuum spectral power was smoothed using two prisms and a spatial filter. The visible light was then polarized using a linear polarizer (10 LP-VIS-B, Newport) and coupled into an optical fiber (SM600, Thorlabs) with an objective (33-438, Edmund Optics). The SM600 fiber was placed in a paddle polarization controller (FPC562, Thorlabs) to allow for further polarization control to maximize interference efficiency. The visible light was then collimated out of the fiber using a fiber port collimator (HPUCO-23-400/700-S-10AC, OZ Optics) and directed toward the sample and reference arm paths using a 50:50 beam splitter (CM1-BS1, Thorlabs). The light in the sample path passed through a short-pass dichroic mirror (DMSP1000R, Thorlabs) where it was combined with the NIR channel. For the NIR portion of the system, a fiber-based interference system could be utilized due to the narrow wavenumber range covered. The NIR light from the super luminescent diode was directed to a 90:10 fiber coupler (TW1300R2A2, Thorlabs) using a fiber circulator (CIR-1310-50-APC, Thorlabs). The coupler ports directed light toward the sample (90 port) and reference arm (10 port) with both ports going through in-line fiber polarization controllers (CPC900, Thorlabs) to optimize the interference efficiency. Light was collimated in the NIR reference arm path using a fiber collimator (F260APC-C, Thorlabs). A second short-pass dichroic mirror with a fine angle adjustment was added to the NIR reference arm path to compensate for the path length effects of the dichroic in the sample arm path. The light was collimated in the NIR sample arm path using an aspheric lens (A397TM-C, Thorlabs). The distance between the aspheric lens and the fiber termination was fine adjusted to allow for co-focusing of the NIR and visible light at the sample. The visible and NIR light were scanned across an objective (440320-9902, Zeiss) using a two-dimensional (2D) galvanometric mirror system (GVS002 TSH25379-X, Thorlabs) to achieve point-wise scanning at the sample. The NIR and visible beams were adjusted to ensure they were coaxial between the dichroic and galvanometric mirror system. Both the visible and NIR reference arms contained glass plates to correct for dispersion as well as neutral density filters to adjust the reference arm power. Visible interference was detected using a custom-built spectrometer. In the spectrometer, the visible light was collimated using a mirror fiber collimator (RC12APC-P01, Thorlabs) and angularly dispersed using a 1200 lines/mm grating (Wasatch). The angularly dispersed light was focused onto a 4096 × 2 pixel line scan camera (spL4096-140 km, Basler) using a custom-built multi-element objective. The visible spectrometer had an axial imaging range of 1 mm in air with an operating bandwidth of 502–705 nm. The NIR interference signal was directed toward a commercial spectrometer (C-1235-1385-GL2K, Wasatch) using the third port of the circulator. The NIR spectrometer had an axial imaging range of 5.7 mm in air, with an operating bandwidth of 1233–1386 nm. (It should be noted that the spectrometer axial imaging range in air is not equivalent to the penetration in tissue. Please refer to Supplementary Information—Penetration Limits of Visible and NIR Bands for mouse ear penetration limits). The sample was moved into focus using a 3D stage (X-XYZ-LSQ150B-K0060-SQ3, Zaber). A custom-built LabVIEW program synchronized the galvanometric mirror scanning with the spectrometer acquisition. The power at the sample was 12.4 mW for visible light and 8 mW for NIR. The system sensitivity was measured to be 93.2 and 104.2 dB for the visible and NIR system, respectively. The visible lateral resolution was 8.8 µm and had an axial resolution of 0.97 µm in tissue. The NIR lateral resolution was 13.8 µm and had an axial resolution of 7.36 µm in tissue. The methods to measure the system sensitivity and resolution are discussed in the Supplementary Information-System Sensitivity and Resolution Measurements section.

### Interferogram preprocessing

Visible and NIR interferograms were preprocessed before calculating the D2-ISOCT metrics. The interferograms were first normalized to the reference arm intensity followed by the DC removal. The data were then resampled into *k*-space (wavenumber space) and digitally compensated for dispersion^42^ before the STFT for the visible and the Fourier transform for the NIR. The visible spectral dependent OCT A-lines, *I*(*λ*,*z*), were normalized to a Rayleigh scattering reference medium and multiplied by a correcting factor of *k*^4^, where *λ* is the center wavelength of the STFT Gaussian window, *z* is the depth, and *k* is the wavenumber (*k* = 2*π*/*λ*). The normalized *µ*_b_ spectra of the samples were calculated by utilizing the relation that *I*^2^(*λ*,*z*) ~ *μ*_b_(*λ*,*z*)^22^_._

### Imaging standards

The Rayleigh scattering reference medium was prepared by diluting an 80 nm polystyrene latex bead solution (Molecular Probes by Life Technologies) to 4% w/v with deionized water. The beads used to characterize the spectral performance (Molecular Probes by Life Technologies; sizes: 100, 370, and 650 nm with coefficient of variations 15%, 3%, and 3%, respectively) were also diluted to 4% w/v with deionized water. Each bead solution was pipetted onto a glass slide before scanning with D2-ISOCT. The normalized bead *µ*_b_ spectra were calculated by analyzing the OCT signal from the top 45 μm of the beads.

Oxygenated and deoxygenated bovine blood samples (Quad Five) used to obtain the sO_2_ calibration spectra were prepared in glass Petri dishes with each containing approximately 10 mL of blood. For the deoxygenated samples, 10% w/v sodium dithionite (Na_2_S_2_O_4_) in phosphate-buffered saline (PBS) was added to the bovine blood at a ratio of 3:1. For the oxygenated samples, the bovine blood was exposed to air for 1 h after adding PBS at a ratio of 3:1. A commercial oxygen probe (MI-730, Microelectrodes, Inc.) measured the blood oxygen pressure (pO_2_) to ensure they were either fully oxygenated and deoxygenated. For the deoxygenated samples, each D2-ISOCT measurement was taken within 10 min of the preparation with Na_2_S_2_O_4_ to avoid oxygenation from air exposure. The normalized blood *µ*_*t*_ was extracted by analyzing the OCT signal from the top 22 microns of the blood samples. The *µ*_*t*_ was calculated according to a simplified OCT backscattering model for sO_2_ quantification (described in Data processing for microvascular sO_2_ measurement)^43^.

The calibration curve for the Doppler blood flow measurements was carried out using a syringe pump (Harvard Apparatus PhD 2000) and plastic micro-tubing (inner diameter 0.353 mm). The flow rates of the bovine blood in the microtube were set by the syringe pump from 3 to 54 µL/min, with intervals of 3 µL/min.

### Animal preparation

All the experimental procedures were approved by the Northwestern University Institutional Animal Care and Use Committee. We monitored the healing process of six ear wounds (two on the left ear and four on the right ear) on one SKH1-Elite (Crl: SKH1-Hrhr) hairless albino mouse for 36 days to demonstrate D2-ISOCT capabilities. We used a 0.35 mm reusable biopsy punch (WPI, World Precision Instruments) to remove cylindrical tissue cores along six different major artery/vein pairs of two ear pinnae, creating six independent wounds. Each wound location was imaged with D2-ISOCT on day 0 (no wound/control), day 1 (wound made), and days 7, 14, 23, 27, 33, and 36 post biopsy punch. For each imaging acquisition, the hairless mouse was first anesthetized with 2.5% isoflurane in 3 standard liter per minute (SLPM) air mixed with pure oxygen for 5 min. After the initial anesthesia, the animal was transferred to an imaging stage and anesthetized with 1.5% isoflurane in 1.5 SLPM air. To ensure the ear was stationary it was attached to a glass slide with double-sided tape. A heating pad was used to maintain the animal’s body temperature.

### Scanning protocols

The visible and NIR bands were simultaneously acquired with each spectrometer collecting 45 000 A-lines/s at an exposure time of 18 µs. Two scanning protocols were used during the data collection.

The first protocol was used for microangiography, sO_2_ measurement, and tissue ultrastructural property analysis. The protocol covered a square FOV of 1.77 × 1.77 mm and was composed of repetitive (4×) unidirectional B-scans of the same cross section. Each B-scan consisted of 400 A-lines per repetition. A total of 512 B-scans × 4 repetitions was sequentially acquired to cover the full FOV, taking a total of 18.2 s to obtain local capillary sO_2_. Single capillary oximetry was carried out by continuously acquiring nine volumetric scans over the same location, equating to a total acquisition time of approximately 170 s.

The second protocol was used for Doppler blood flow measurements, covered a FOV of 1.77 × 1.77 mm and was composed of repetitive counter-directional B-scans of the same cross section. Each B-scan consisted of 2000 A-lines for each direction. A total of 256 B-scans × 2 (for bidirectional scanning) were sequentially acquired to cover the full FOV, taking a total of 22.8 s.

The large FOV image seen in Fig. 2 was collected by utilizing scanning protocols 1 and 2 over a 4 by 4 mosaic, increasing the FOV to approximately 6.0 × 6.5 mm. The imaging site was shifted using the mechanical stage first horizontally using LabVIEW software and then vertically with manual adjustments. We allowed some overlap between adjacent scans for better stitching of individual images of the mosaic. The total time for the large FOV under protocol 1 was ~4.8 min and under protocol 2 was ~6.1 min.

### Data processing for microangiography

The sweeping Gaussian window for visible microangiography sampled the spectrum into 18 equally spaced narrow bands in k-space. The swept wavelength range was 507–702 nm, and the size of the Gaussian window was 0.35/µm. This gave a STFT bandwidth of 20.3 nm at 603 nm and relaxed the axial resolution to 7.88 µm. For the NIR microangiography, the size of the Gaussian window was 0.37/µm and covered a wavelength range of 1233–1386 nm. This gave a STFT bandwidth of 100 nm at 1309.5 nm and relaxed the axial resolution to 7.55 μm. Angiography contrast originates from the decorrelation between repetitive B-scans due to the sample movement found in blood vessels. Therefore, bulk sample movement must be mitigated to increase the contrast of blood vessels. We adopted a three-step digital approach to correct for the bulk sample movement^44^. First, four B-scans of the same cross-sectional area were co-registered according to their cross-correlation functions. The axial global phase fluctuations between the four scans were then corrected with two phase-modifiers. Finally, the motion-corrected data were obtained by calculating the expected value of the amplitude between the four scans. The 3D angiography data were then averaged spatially and across different STFT windows to reduce the background noise. The visible and NIR *en face* microangiography images had values set to zero, which were <~1.5 times their mean intensity. The 2D binary mask used in the *D* calculations was obtained by thresholding the visible *en face* microangiography to ~1.5 times its mean from the surface to 150 μm. The 3D microangiography binary mask used in sO_2_ calculations was obtained by thresholding each visible microangiography cross section to ~1.5 times the mean intensity from the surface to 150 μm in depth.

### Data processing for microvascular sO_2_ measurement

The *μ*_*t*_ of blood vessels were extracted according to the following simplified OCT backscattering model for sO_2_ quantification^43^. In this model, the intensities for the hemoglobin absorption bands (520–610 nm) along the axial direction of the OCT A-line separated by a distance *z* can be related to each other through *μ*_*t*_:

4 $$I\left( {\lambda ,z_0 + z} \right)^2 = C\rho I\left( {\lambda ,z_0} \right)^2e^{ - 2\mu _t(\lambda )z}$$

where *C* is a constant, *ρ* is the local reflectance, and *I*(*λ*,*z*_0_) is the OCT A-line intensity at depth *z*_0_. We simplified *μ*_*t*_(*λ*) as:

5 $$\mu _t(\lambda ) = a\left( {g\left( \lambda \right)} \right) \times \mu _{\mathrm{s}}(\lambda ) + \mu _{\mathrm{a}}(\lambda )$$

where *a* is a function of the hemoglobin anisotropic factor *g*, and *μ*_s_ and *μ*_a_ are the scattering and absorption coefficients of blood, respectively. This simplified relation was verified using Mie theory for the attenuation coefficients of oxygenated and deoxygenated blood, as shown in Fig. 1d. According to (4), the *µ*_*t*_ of a blood vessel could be written as the following, where *z*_0_ is the depth location for the top of the blood vessel, and *z* is the axial length of the blood vessel on the thresholded visible angiogram:

6 $$\mu _t\left( \lambda \right)\sim \frac{1}{2}{\mathrm{log}}\left( {\frac{{I\left( {\lambda ,z_0} \right)^2}}{{I\left( {\lambda ,z_0 + z} \right)^2}}} \right){\mathrm{/}}z$$

Provided there is sufficient signal across the spectrum at the measured blood vessel, it can be seen in (6) that the normalized *μ*_*t*_ used in calculating sO_2_ can be extracted regardless of scattering above the vessel. This is because (6) only considers the contrast in the spectrum through the vessel’s axial length on the angiogram mask.

The *I*(*λ*,*z*) used in calculating sO_2_ with the above relation was generated using an STFT Gaussian window size of 0.91/μm. The windows were equally spaced in wavenumber from approximately 520 to 610 nm, totaling 18 windows. This produced a measured axial resolution of 8.5 μm in tissue, as shown in the Supplementary Information-System Sensitivity and Resolution Measurements section. To calculate the *μ*_*t*_ of blood vessels, their spatial location needed to be identified. This was accomplished using the threshold microangiography generated by the visible windows as described in the Materials and methods—Data processing for microangiography section. The microangiography STFT windows were not the same as the sO_2_ STFT windows because the additional windows from 610 to 702 nm helped improve the signal-to-noise ratio. Furthermore, a smaller window size was necessary to prevent low-pass filtering the sO_2_ contrast from the spectra. However, the sO_2_ STFT windows could still sense capillaries, as shown in the Supplementary Information-sO_2_ STFT Window Capillary Sensing section.

To isolate regions for spatial spectral averaging, lateral regions of interest (ROIs) were manually drawn on the *en face* projective visible microangiography to select a single arteriole/venule or a local microvascular network. Then, a 3D ROI was generated by extruding the manually selected lateral ROI along depth, corresponding to a thickness of ~150 μm from the skin top surface. Next, blood vessels were segmented from this 3D ROI for each microangiographic wavelength band using a threshold-based algorithm. In each A-line of the 3D microangiography, the top and bottom of the elliptically shape vessels were calculated. Blood vessel *μ*_*t*_ in this ROI was then calculated by setting *z* in (6) to the mean segmented vessel size. The local capillary sO_2_ in the ROI was then generated using least squares fitting for the vessel *μ*_*t*_ to the sO_2_ calibration curves.

The sO_2_ of a single capillary (demonstrated in Fig. 3a, day 36) was calculated using the same method, except that *I*(*λ*,*z*) was obtained by averaging OCT signals over nine consecutive volumetric scans. This resulted in a total temporal average of 170 s. Nine consecutive volumetric scans were co-registered according to their cross-correlation functions before averaging to remove the possible movement of the animal between scannings.

Example spectra of blood vessels and their quantified sO_2_ fitted spectra are shown in the Supplementary Information-Microvascular Spectra and sO_2_ section.

### Data processing for microvascular flow rate measurement

Flow calculations assumed consecutive A-lines to be at the same location due to the high density of A-lines per B-scan. Thus, the phase shifts between any two consecutive A-lines was proportional to the blood flow velocity projected along the beam axis. The Doppler phase images were obtained by taking the first derivative of the phase of complex OCT B-scans. When phase wrapping was observed, a correction was applied to resolve any 2*π* ambiguity. To reduce the phase shifts caused by the instrumental raster scan, each pair of counter-directional phase shift B-scans were averaged.

In this work, the absolute blood flow rate of vessels was calculated by multiplying a vessel’s *x*–*y* plane (plane orthogonal to the beam axis) displayed cross-sectional area, *A*_disp_, with its corresponding projective flow velocity,*V*_p_. The principle flow rate, *F*, can be calculated using (7) where *A* is a vessel’s cross-sectional area and *v* is the flow velocity:

7 $$\begin{array}{*{20}{c}} {F = vA} \end{array}$$

*v* can be related to *V*_p_ through (8) and *A* can be related to *A*_disp_ through (9):

8 $$\begin{array}{*{20}{c}} {v = \frac{{V_{\mathrm{p}}}}{{{\mathrm{cos\theta }}}}} \end{array}$$

9 $$\begin{array}{*{20}{c}} {A = A_{{\mathrm{disp}}}{\mathrm{cos}}\theta} \end{array}$$

where *θ* is the angle between the blood vessel axis and the beam axis^45^. It can be seen that the cosine term cancels out when calculating *F* from *V*_p_ and *A*_disp_ and, therefore, the angle does not need to be considered. The *en face* projective flow maps were produced by assigning each pixel to the mean flow rate of all vessel segments projected onto this pixel from the surface to a depth of 300 μm. *A*_disp_ was measured by binarizing the absolute value of the phase shift intensity thresholded at *π*/50, while *V*_p_ was related to the phase shift from the calibration shown in Fig. 1e.

### Data processing for microvascular drO_2_ estimation

To estimate the mouse ear microvascular drO_2_ during the wound-healing response, we calculated the oxygen consumption (gas volume of the oxygen consumed per unit time) of major blood vessels^18^ according to the blood flow rate and sO_2_ measured by D2-ISOCT. We obtained an *en face* projection map of drO_2_ according to:

10 $${\mathrm{drO}}_2 = 1.34 \times C_{{\mathrm{Hb}}} \times F \times {\mathrm{sO}}_2$$

where 1.34 is the oxygen-binding capacity of hemoglobin (mL/g), *C*_Hb_ is 0.13 gm/L as the total hemoglobin concentration^46^, *F* is the microvascular blood flow rate of the major blood vessels, and sO_2_ is the approximate oxygen saturation of major blood vessels.

### Data processing for microvascular mrO_2_ estimation

To estimate the mouse ear microvascular mrO_2_ during the wound-healing response, we calculated the oxygen consumption difference between the arteriole and the venule^18^ according to their upstream mean blood flow rates and sO_2_ measured by D2-ISOCT:

11 $${\mathrm{mrO}}_2 = 1.34 \times C_{{\mathrm{Hb}}} \times (\bar F_{{\mathrm{arteriole}}} \times \overline {{\mathrm{sO}}_2} _{{\mathrm{arteriole}}} \\ - \bar F_{{\mathrm{venule}}} \times \overline {{\mathrm{sO}}_2} _{{\mathrm{venule}}})$$

where *F*¯_arteriole_ and *F*¯ _venule_ are the upstream mean flow rates of the arteriole and venule, respectively, and $\overline {{\mathrm{sO}}_2} _{{\mathrm{arteriole}}}$ and $\overline {{\mathrm{sO}}_2} _{{\mathrm{venule}}}$ are the upstream mean sO_2_ of the arteriole and venule, respectively.

### Pericapillary ultrastructural sensing

Pericapillary ultrastructural sensing was conducted by quantifying *D* from the directly measured visible STFT A-line intensity. In-depth details of how *D* can be related to the STFT A-line intensity are provided in the Supplementary Information- Approach for Obtaining Shape Factor *D* section. STFT A-lines used in the ultrastructural sensing were obtained using a Gaussian window size of 0.49/μm, which sampled the visible spectrum into 13 equally spaced narrow bands in *k*-space. The swept wavelength range was 507–702 nm. This gave an STFT bandwidth of 26.8 nm at 585.5 nm and relaxed the axial resolution to 12.6 μm. Normalized *μ*_b_ was first calculated using a STFT according to the above sweeping Gaussian window, followed by a least-square fitting to obtain *D* from the relationship that *μ*_b_ ~ *k*^4−*D*^. This relationship assumes that the tissue is a continuously random media^21,23^. The *D* values from blood vessels were excluded using the microangiography mask and the average *D* value for the pericapillary space was calculated from 90 to 200 µm in depth for each FOV. The *en face* Δ*D* projection maps (average of Δ*D* along depth from 90 to 200 µm) were shown with their value being the difference from their FOV average *D* value.

To illustrate the process of calculating *D* values from the spectra of nonvascular tissues, visible OCT measured spectra of nonvascular tissues and their *D*-fitted spectra are shown in Supplementary Information- Tissue Spectra and *D* Values. Example spectra are from nonvascular tissues within large and small ROIs, respectively.

### Statistical analysis of ultrastructure during wound healing

D2-ISOCT wound recovery monitoring was statistically investigated by performing a one-tailed Student’s *t*-test on the Δ*D* values from the pre-wounded (control) and wounded pericapillary tissue. The Δ*D* in the wounded sites was calculated from a manually placed ROI circle approximately 0.8 mm in diameter around the wound. The results are presented as the mean ± SEM. In principle, the granulation tissues formed during wound recovery consisted of highly disorganized collagen with higher intensities compared with normal non-invasive skin, leading to higher Δ*D*. Thus, we applied a one-tailed Student’s *t*-test for five or six independent wounds and calculated their *p* values at different time points from comparison with non-invasive control.

## Electronic supplementary material

Supplementary Information
