# Supplementary material for: Single capillary oximetry and tissue ultrastructural sensing by dual-band dual-scan inverse spectroscopic optical coherence tomography
Source: Light Sci Appl. 2018 Aug 29;7:57. doi: 10.1038/s41377-018-0057-2 (PMC6113297; doi:10.1038/s41377-018-0057-2)
Supplement: Supplementary file 1 — Supplementary Information [file 41377_2018_57_MOESM1_ESM.docx]

**Title: Single capillary oximetry and tissue ultrastructural sensing by dual-band dual-scan inverse spectroscopic optical coherence tomography**

**Running title:**

**OCT functional capillary-level microangiography**

**Authors:**

Rongrong Liu†^，1^ ([rongrongliu2019@u.northwestern.edu](mailto:rongrongliu2019@u.northwestern.edu)),

James A. Winkelmann†^，1^ ([jameswinkelmann2014@u.northwestern.edu](mailto:jameswinkelmann2014@u.northwestern.edu)),

Graham Spicer^，2^ ([grahamspicer2013@u.northwestern.edu](mailto:grahamspicer2013@u.northwestern.edu))

Yunxiao Zhu^，1^ ([yunxiaozhu2014@u.northwestern.edu](mailto:yunxiaozhu2014@u.northwestern.edu))

Aya Eid^，1^ ([aeid@u.northwestern.edu](mailto:aeid@u.northwestern.edu))

Guillermo A. Ameer^，1^ ([g-ameer@northwestern.edu](mailto:g-ameer@northwestern.edu))

Vadim Backman^，1^ ([v-backman@northwestern.edu](mailto:v-backman@northwestern.edu))

Ji Yi*^, 3^ ([jiyi@bu.edu](mailto:jiyi@bu.edu), Tel.: (617) 638 6599)

**† These authors contributed equally to this paper.**

* **Corresponding Author**

**Affiliations:**

^1^Department of Biomedical Engineering, Northwestern University, Evanston, Illinois 60208, USA

^2^Department of Chemical and Biological Engineering, Northwestern University, Evanston, Illinois 60208, USA

^3^Department of Medicine, Boston University School of Medicine, Boston, Massachusetts 02118, USA

**Supplementary Information**

Figure S1: D2 ISOCT Benchtop System Schematic. Reference Mirror (MR). Neutral Density Filter (NDF). Dispersion Compensation (DC). Mirror (M). Diffraction Grating (DG). Dichroic Mirror (DM). Visible Collimator (VC). Beam Splitter (BS). Spatial Filter (SF). Prism (P1 & P2). Polarization Controller (PC). NIR Collimator (NIRC). Diffraction Grating (DG).

**System Sensitivity and Resolution Measurements**

The illumination intensity of the sample was 12.4 mW and 8 mW for visible and NIR channel, respectively. Since OCT performs Fourier transform, higher frequencies corresponding to deeper depths in the sample are limited to spectrometer resolution and thus result in a sensitivity roll-off. Therefore, the system was characterized by measuring the impulse response function of a reflection off a mirror vs. imaging depth, as shown in Figure S2. Impulse response was calculated for the visible and NIR channel by attenuating illumination at the mirror by 27.5 dB. The sensitivity was then calculated by measuring the height of the second peak with respect to the noise floor plus the round-trip attenuation of 55 dB. The sensitivity for the visible and NIR channel was 93.9 dB and 104.2 dB, respectively. Axial resolution for the full spectral ranges and sO_2_ short time Fourier transform (STFT) axial resolution was determined by the impulse width at – 6 dB from the height of the second peak with respect to the noise floor. Visible axial resolution was 1.3 $\mu m$ in air corresponding to an axial resolution of .97 $\mu m$ in tissue, while NIR was 9.8 $\mu m$ in air corresponding to an axial resolution of 7.36 $\mu m$ in tissue. The visible sensitivity roll-off was – 15 dB mm^-1^, while the NIR roll-off was – 3.5 dB mm^-1^. sO_2_ STFT axial resolution in air was an average of 11.32 $\mu m$ corresponding to an axial resolution of 8.5 $\mu m$ in tissue. Lateral resolution was determined by scanning a razor blade and measuring the edge response. The lateral resolution was found to be 8.8 $\mu m$ and 13.8 $\mu m$ for visible and NIR, respectively.

Figure S2: System sensitivity and axial resolution report. The roll-off impulse response of a mirror vs. depth for the (a) visible and (b) NIR channels. The full visible spectral range resolution and sensitivity was 1.3 $\mu m$ and 93.9 dB, respectively at the second peak. The full NIR spectral range resolution and sensitivity was 9.8 $\mu m$ and 104.2 dB, respectively at the second peak. (c) The air axial resolution of the sO_2_ STFT windows measured from the second peak in (a). The average window resolution was 11.32 $\mu m$ (shaded region is standard error of the mean over 1000 A-lines).

**Penetration Limits of Visible and NIR Bands**

The imaging penetration limits of D2-ISOCT into the mouse ear were quantified for the visible and NIR bands. Spectrally dependent OCT B-scans of the mouse ear before injury and the corresponding mean A-line intensity profile are shown in Figure S3. For the visible channel three sO_2_ STFT windows were analyzed with center wavelengths at 520.4 nm, 558.6 nm, and 608.9 nm. The imaging penetration limits in Figure S3 were defined as 5% the maximum A-line intensity after noise floor subtraction. The noise floors were calculated as the mean intensities of signals at a depth range of 537.6 to 582.4 μm from the skin surface for the visible channel, and at a depth range of 627.2 to 672.0 μm from the skin surface for the infrared channel. As expected due to scattering, 520.4 nm had the worst penetration with an imaging limit of approximately 241.9$\mu m$ and defines the sO_2_ imaging limit of D2-ISOCT for the mouse ear. The NIR band had an imaging limit of approximately 528 $\mu m$ providing it with the ability to have high signal-to-noise-ratio abilities in measuring the flow rates of deep vessels as shown in Figure S9. Within the sO_2_ imaging limit (241.9$\mu m$), signals from both channels are valid.

Figure S3: Spectrally dependent OCT B-scans and their corresponding average A-line intensity for the mouse ear before the injury. (a) – (c): B-scans of ear generated by sO_2_ STFT windows with central wavelength of 520.4 nm (a), 558.6 nm (b), 608.9 nm (c). (d) NIR B-scan. (e) – (h) shows (a) – (d) shifted to the top. (i) – (l): Average A-line intensity of B-scans shown in (e) – (h). All intensities in (i) – (l) are after noise floor subtraction and normalized by its maximum. The penetration limit is defined as 5% of the maximum intensity in (i) – (l). Penetration Limits: 520.4 nm (241.9 μm), 558.6 nm (353.9 μm), 608.9 nm (439.1 μm), NIR (569.0 μm). Scale bar: 100 μm.

**Comparison of NIR and Visible Microangiography**

The comparison of the visible and NIR microangiography can be seen in Figure S4 and Figure S5. Figure S4 shows a microangiography cross-section where the higher penetration of the NIR angiography can be seen. Figure S5 shows *en face* microangiography for the same field of view shown in Figure 3 Day 0 from 0 – 100 $\mu m$, 100 – 200 $\mu m$, and 200 – 300 $\mu m$ in depth. For the superficial layer (0 – 100 $\mu m$) in Figure S5a and Figure S5d it can be seen that the visible microangiography had better resolution in resolving the capillary network compared to the NIR. Also, it can be seen in Figure S5b that the arterioles and venules analyzed in Figure 3 could be resolved by visible microangiography allowing the visible channel to calculate sO_2_ from them as shown in Figure S7b. Figure S5 also shows how the NIR produced greater signal-to-noise ratio for the arteriole and venules which was necessary in measuring Doppler phase shifts for flow measurements.

Figure S4: Microangiography cross-sections on Day 0 of the mouse ear for (a) visible and (b) NIR. Scale bar: 100 $\mu m$.

Figure S5: *En face* microangiography projections of visible (a – c) and NIR (d – f) bands of the field of view shown in Figure 3 Day 0. Depth selection (a,d) 0 – 100 $\mu m$; (b,e) 100 – 200 $\mu m$; (c,f) 200 – 300 $\mu m$. Scale bar: 100 $\mu m$.

Figure S6: The structural cross section of a mouse ear wound on Day 14 after the ear punch. (a) Visible channel; (b) NIR channel. EP: epidermis; DE: dermis; GT: granulation tissues. Yellow lines outline Dermis/Granulation Tissue layer. Scale bar: 100 $\mu m$.

Figure S7: Microvascular sO_2_, blood flow rates, drO_2_, and tissue ultrastructural properties of a SKH1-Elite hairless albino mouse ear during wound healing on Day 0 (no wound/control), Day 1 (wound made), Day 7, Day 14, Day 23, Day 27, Day 33 and Day 36 post biopsy punch. (a) *En face* projections of superficial (0 – 100 $\mu m$) microvascular sO_2_ during wound healing. The white box on Day 36 crops a capillary segment for which sO_2_ was calculated. (b) *En face* projections of dermal (100 – 200 $\mu m$) arteriolar and venular sO_2_ during wound healing. (c) *En face* projections of blood flow rates during wound healing. (d) *En face* projections of drO_2_ during wound healing. (e) *En face* projections of ΔD during wound healing. The depth range to calculate D was 90 – 200 μm from the skin surface. The white dashed circles indicate the regions around the wound in (a) – (e). Scale bars: 200 μm.

**sO_2_ STFT Window Capillary Sensing**

Applying a STFT to the interferogram to get spectroscopic information does result in a degradation to image axial resolution as noted by the Figure S2c. However, this does not eliminate the ability for individual sO_2_ STFT windows to be sensitive to capillary-level information. Evidence of this supported by angiogram capillary signal being observed across all sO_2_ STFT windows. Angiogram *en face* and cross-sectional images from Figure 3 (a) Day 0 generated using the sO_2_ STFT windows can be seen in Figure S8a – Figure 8Sd. Figure 8Sd shows a cross-section blow-up of two capillaries with their depth resolved intensity profile shown in Figure S8e and Figure S8f. Only the first peak of the depth resolved profile is considered as noted by the green dotted lines and the second angiogram peak of vessels was not considered for sO_2_ masks. For capillaries C1 and C2 their full width at half maximum (FWHM) from the full spectrum angiogram was 41 $\mu m$ and 48 $\mu m$, respectively. It can be seen in Figure S8g and Figure S8h that axial angiogram vessel FWHM intensity only varies slightly across angiograms generated using individual sO_2_ STFT windows. This ensures that all STFT windows were sensitive to capillaries and they experienced a similar axial resolution in biological tissue. The resulting spectra of various vessel types is shown in Supplementary Information- Microvascular Spectra and sO_2_.

Figure S8: Angiogram analysis using the sO_2_ STFT windows. (a) *En face* projection of Figure 3 (a) Day 0 using all sO_2_ STFT windows. The white vertical line marks a B-scan as B_0_, which crosses two capillaries within the green box. The two capillaries are labelled as C1 and C2 in the blow-up of the green box. Scale bar: 100 $\mu m$. (b) Angiogram cross section of B_0_ using all sO_2_ STFT windows. The green box crops C1 and C2. Scale bar: 100 $\mu m$. (c) The angiogram shown in (b) with the skin surface shifted to the top of the image. Scale bar: 100 $\mu m$. (d) A blow up of the green box in (c). Scale bar: 10 $\mu m$. (e) – (f) The spectral depth resolved intensity profile of C1 (e) and C2 (f) from the skin surface, respectively. The green dashed lines indicate the depth range, 40 – 110 $\mu m$ from the skin surface, to calculate FWHM. Only the first angiogram vessel peak was considered. Error bar = S.E.M. (g) – (h) The FWHM of the depth resolved A-line intensity profile for C1 (g) and C2 (h). Blue line: FWHM calculated for each STFT window. Red line: FWHM calculated from angiography averaged over all sO_2_ STFT windows.

**Microvascular Spectra and sO_2_**

To illustrate the process of sO_2_ calculation, we select five regions of interest (ROI): an arteriole, a large-size capillary network, a medium-size capillary network, a small-size capillary network, and a single capillary. We first show their *en face* projective microvascular masks for signal segmentation in Figure S9a – Figure S9e. Then to extract the spectra according to equation (6), the logarithmic spectral intensities from above and below the blood vessels are shown in Figure S9f – Figure S9j. Also, shown is their difference which reveals the sO_2_ contrast in the signal. Through least square fitting the normalized difference, or normalized µ_t_, to the sO_2_ calibration curves, the sO_2_ for the segmented vessel(s) can be calculated as shown in Figure S9k – Figure S9o. The sO_2_ results approximately match the *en face* sO_2_ map shown in Figure 3(a) Day 0. The discrepancies are attributed to the fact that the sO_2_ of each pixel in Figure 3(a) was calculated by averaging the results from multiple ROIs covering that pixel.

Figure S9: Example sO_2_ calculations. *En face* projections of segmenting blood vessels: (a) an artery, (b) – (d) capillary networks, (e) a single capillary. (a) – (d) is field of view from Figure 3 Day 0 and (e) is capillary blow up on Figure 3 Day 36. Scale bar: 100 $\mu m$. (f) – (j) The logarithmic spectral intensities from the top (red) and bottom (blue) of blood vessels in (a) – (e) and their differences (black) used in sO_2_ fitting. (k) – (o): Comparisons between the measured spectra (black) of blood vessels in and their sO_2_ fitted spectra (green). The calibration spectra for oxygenated (red) and deoxygenated (blue) conditions are also shown for reference.

**Approach for Obtaining Shape Factor *D***

If tissue is assumed to be a continuously varying refractive index medium, its refractive index autocorrelation function, $B_{n}$, between any two points separated by a distance, $r$, can be modeled by the following^1^:

$B_{n}\left( r \right)=A_{n}\left( \frac{r}{l_{c}} \right)^{\frac{D-3}{2}}K_{\frac{D-3}{2}}\left( \frac{r}{l_{c}} \right)$	(S1)

where $A_{n}$ is the amplitude of refractive index fluctuations, $l_{c}$ is a length scale parameter, $K_{\{.\}}$ is the modified Bessel function of the second type, and *D* is the functional “shape factor” describing what kind of function $B_{n}$ is. When *D* is between 0 and 3, $B_{n}$ is a power law and tissue is organized into mass fractals. *D* values between 3 and 4 results in $B_{n}$ having a stretched exponential shape, and as *D* approaches infinity $B_{n}$ is a Gaussian. Using the Gladstone-Dale relationship, $B_{n}$ can be found to be proportional to the physical spatial distribution of macromolecular density. Therefore, physical tissue distributions on the nanoscale can be characterized by measuring tissue optical properties.

By taking the Fourier transform of $B_{n}$ and using the first-order Born approximation the differential scattering cross-section, $\sigma$, of tissue can be obtained^1^:

$B_{n}\underset{\Leftrightarrow}{F.T.}\sigma\left( \theta,\phi\right)=A_{n}\pi^{-\frac{1}{2}}2^{\left( \frac{D}{2}-\frac{3}{2} \right)}\Gamma\left( \frac{D}{2} \right)k^{4}l_{c}^{3}\frac{1-{sin}^{2} \left( \theta\right){cos}^{2} \left( \phi\right)}{\left\{ 1+\left[ 2kl_{c}\sin\left( \frac{\theta}{2} \right) \right]^{2} \right\}^{2}}$	(S2)

where $\left( \theta,\phi\right)$ are the polar and azimuthal angles, $k$ is the wave number, and $\Gamma$ is the gamma function. By integrating over all azimuthal angles, evaluating $\theta$ in the backwards scattering direction ($\theta=\pi$), and assuming tissue to be highly forward scattering (${kl}_{c}$>>1), the backscattering coefficient, $\mu_{b}$, can be related to refractive index autocorrelation function parameters through the following^1^:

$\mu_{b}(k)=A_{n}2^{\left( \frac{D}{2}+\frac{1}{2} \right)}\pi^{\frac{3}{2}}\Gamma\left( \frac{D}{2} \right)l_{c}^{3-D}k^{4-D}$	(S3)

Now the simplified relationship $\mu_{b}\left( k \right)$~$k^{4-D}$can be drawn.

$\mu_{b}(k)$ at each OCT image voxel can be related to the STFT A-line intensity, $I (k, z)$ through the following^1^:

$\mu_{b}(k,z)=\frac{4\pi I^{2}\left( k,z \right)}{rI_{0}^{2}\left( k \right)L\left( k \right)}exp(2z\mu_{s}(k))$	(S4)

where $z$ is the depth location, $r$ is the reflectance of the reference arm, $I_{0}$ is the STFT window illumination intensity, $L$ is the temporal coherence length of the STFT window, and $\mu_{s}$ is the bulk tissue scattering coefficient. Therefore, after obtaining the STFT A-line intensity, the shape of $\mu_{b}$ can be obtained using the simplified relation that $I^{2}\left( k, z \right)$ ~ $\mu_{b}\left( k, z \right)$. Now *D* can be spatially obtained for each OCT imaging voxel by using the simplified relation that $\mu_{b}\left( k \right)$~$k^{4-D}$.

**Tissue Spectra and *D* Values**

To illustrate the process of calculating *D* values, we select two example spectra of nonvascular tissue within a large ROI and a small ROI from our processing. Their *en face* projective nonvascular masks for signal segmentation are shown in Figure S10a and Figure S10b. Their spectra are shown in blue in Figure S10c and Figure S10d with their *D*-fitted spectra shown in red as comparison. It can be seen that the nonvascular tissue spectra follows a monotonic decrease in scattering with increasing wavelength, as has been previously noted^1^.

Figure S10: Example spectra of non-vascular tissues and their *D*-fitted spectra. (a) and (b): *En face* projections of non-vascular masks within different ROIs used in *D* value calculation. Scale bars in (a) and (b): 100 $\mu m$. (c) and (d) The measured (blue) and *D*-fitted (red) spectra of non-vascular tissues in (a) and (b), respectively.

**D2-ISOCT Doppler Measurements**

Figure S11: Doppler phase shift measurements from Figure 3 Day 0. (a) Visible *en face* angiography, Depth Range: 0 – 150 $\mu m$. (b) NIR *en face* angiography, Depth Range: 0 – 375 $\mu m$. (c) Visible Doppler phase shift cross section marked by the horizontal green line in (a). (d) NIR Doppler phase shift cross section marked by the horizontal green line in (b). Scale bar: 100 $\mu m$.

**References**

1.	Yi, J. *et al.* Spatially resolved optical and ultrastructural properties of colorectal and pancreatic field carcinogenesis observed by inverse spectroscopic optical coherence tomography. *J. Biomed. Opt.* **19,** 36013 (2014).
